# Isobaric tags for relative and absolute quantification-based proteomic analysis of testis biopsies in rhesus monkeys treated with transient scrotal hyperthermia

**DOI:** 10.18632/oncotarget.20719

**Published:** 2017-09-08

**Authors:** Meng Rao, Sha Ma, Shifu Hu, Hui Lei, Yanqing Wu, Yanfei Zhou, Wei Xia, Changhong Zhu

**Affiliations:** ^1^ Family Planning Research Institute, Tongji Medical College, Huazhong University of Science and Technology, Wuhan, China; ^2^ Department of Reproduction and Genetics, The First Affiliated Hospital of Kunming Medical University, Kunming, China; ^3^ Wuhan Women and Children's Health Care Center of Hubei Province, Wuhan, China; ^4^ Changsha Hospital for Maternal and Child Health Care, Changsha, China; ^5^ Reproductive Medicine Center, Tongji Medical College, Huazhong University of Science and Technology, Wuhan, China

**Keywords:** heat stress, spermatogenesis, rhesus monkeys, iTRAQ, proteomics

## Abstract

This study aimed to examine the cellular and molecular events that occur in rhesus monkey testes after scrotal hyperthermia. Eight male adult rhesus monkeys were subjected to scrotal hyperthermia at 43°C for 30 min daily for 6 consecutive days. Sperm concentration, reproductive hormones, and testis histology were examined before hyperthermia (day 0), and at 8, 15, 30, 45, 60, 75, and 90 days after the initiation of hyperthermia. iTRAQ-based proteomic analysis was conducted on testicular tissues collected on days 0, 8, and 60 to identify differentially expressed proteins at the early and recovery stages of testicular damage. The sperm concentration was significantly decreased at days 30 and 45 after treatment (*p* < 0.01) and recovered to baseline at day 60. When compared with day 0, 101 and 24 differentially expressed proteins were identified at days 8 and 60 after heat treatment, respectively. The molecular functions of the differentially expressed proteins at day 8 were mainly nucleic acid binding, unfolded protein binding, nucleotide binding, and nucleoside phosphate binding. Spliceosome was enriched as the most significant pathway at day 8. CIRBP, PSIP1, Sam68, and Decorin were validated and found to be consistent with the proteomic data, indicating the reliability of the proteomic profiles identified in this study. In summary, we suggest that the proteins identified in this study may play important roles in heat-induced spermatogenic impairment. Some of these proteins, such as CIRBP, PSIP1, Sam68, and Decorin, may be early molecular targets responsible for spermatogenesis suppression induced by heat treatment.

## INTRODUCTION

In most mammals, including humans, the testes are located in the scrotum outside the main body cavity to maintain a lower-temperature environment for normal spermatogenesis [1]. However, the testicular thermoregulation balance can be disrupted by several endogenous and exogenous factors. Cryptorchidism and varicocele are the main endogenous factors that lead to testicular hyperthermia and affect normal spermatogenesis [2, 3]. Scrotal heat stress from occupational exposure to high environmental temperatures, such as that experienced by welders and drivers, can have a deleterious effect on spermatogenesis [4, 5].

Hot tubs are popular in China and can be enjoyed in various places, including hotels, holiday resorts, scenic spots, and even at home [6]. Most hot tub enthusiasts are attracted by commercial advertisements that exaggerate the health effects of hot tubs, with no concern for the potential damage to reproduction. The temperature in a hot tub generally varies from 37°C to 45°C, which is much higher than the normal scrotal temperature. In our previous clinical study, we simulated a hot tub environment and exposed healthy adult volunteers to scrotal hyperthermia at 43°C ten times for 30 min each, once daily or once every 3 days [6, 7]. We found an obvious decrease in sperm concentration, total sperm count, sperm motility, and sperm function after scrotal hyperthermia. Sperm DNA integrity was also severely damaged [6, 7]. Studies have indicated that heat stress–induced spermatogenic impairment is mainly due to mitochondria-dependent germ cell apoptosis [6, 8, 9]. Nevertheless, the complicated process of apoptosis is regulated by many factors, and the molecular mechanisms that underlie this process are still largely unknown.

Rocha et al. [10] investigated the effect of testicular hyperthermia on ram semen plasma proteome using two-dimensional SDS-PAGE and mass spectrometry (2DE-MS) and found a series of protein targets involved in sperm protection, maturation, and fertilization. Another two studies have revealed the molecular mechanism of heat-induced spermatogenic impairment in mouse and human testes, respectively [11, 12]. Both studies were based on 2DE-MS techniques, which are less sensitive and less effective than the isobaric tags for relative and absolute quantification (iTRAQ) -based proteomic method for identifying specific proteins, very small or large proteins, proteins expressed at very low levels, and membrane proteins [13, 14]. In current proteomics research, the method used to quantify proteins has developed into a combination of iTRAQ and liquid chromatography–tandem mass spectrometry (LC–MS/MS) [15]. iTRAQ has relatively high throughput and simultaneously provides information on protein quantitation and identification [13, 14]. The aim of this study was to use iTRAQ proteomic technology to analyze the proteins expressed in rhesus monkey testes at the early stage of spermatogenic damage and at the recovery stage after scrotal hyperthermia. Some key differentially expressed proteins and potential pathways were revealed for further study to clarify the underlying mechanisms involved in heat stress–induced spermatogenic impairment.

## RESULTS

### Sperm parameters and reproductive hormone levels

**Figure 1:**
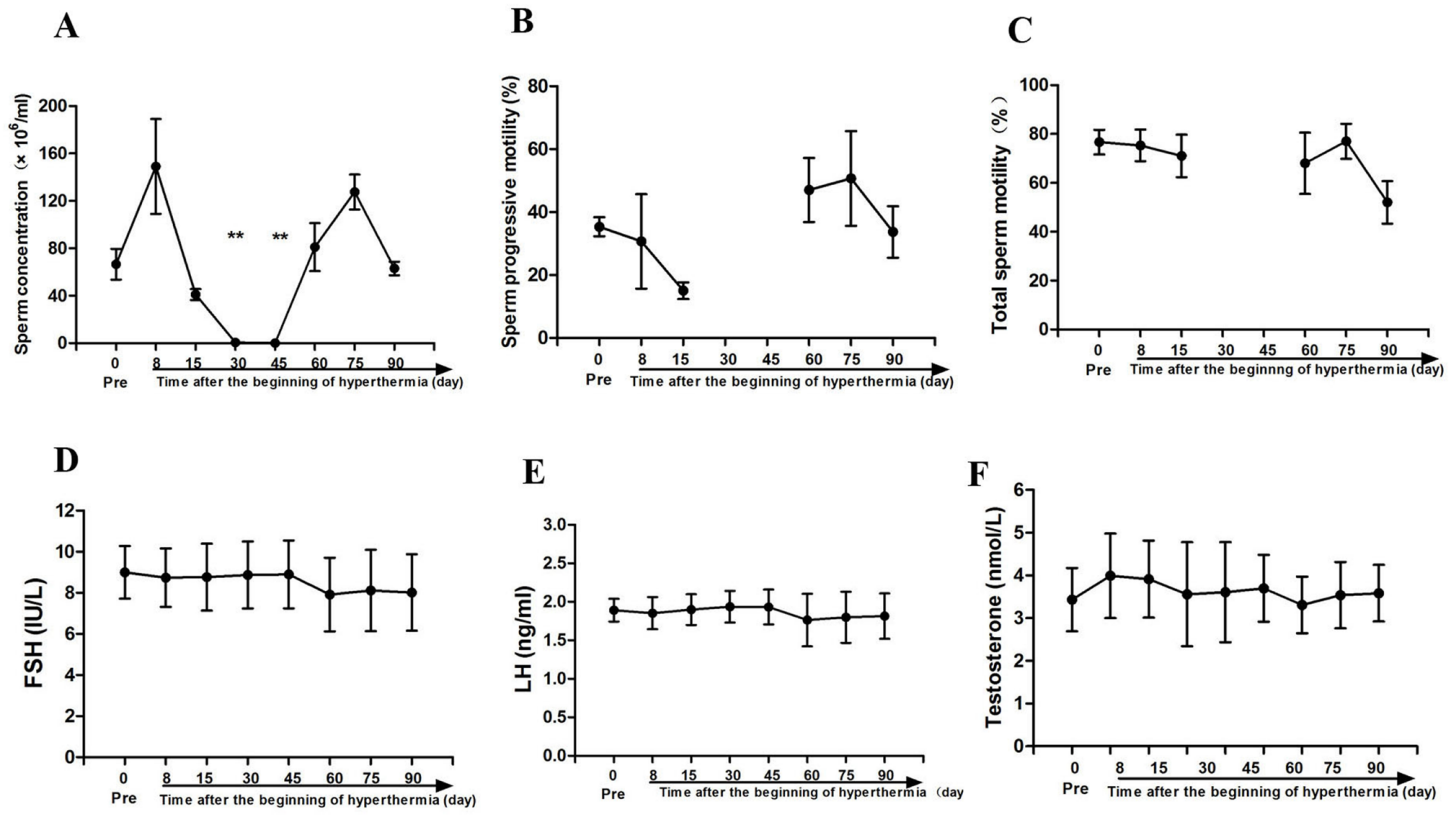
Sperm parameters and reproductive hormone levels before scrotal hyperthermia and at different time points after hyperthermia **(A)** sperm concentration; **(B)** progressive sperm motility; **(C)** total sperm motility; **(D, E** and **F)** represent FSH, LH, and total testosterone levels, respectively. N=3 for sperm parameter analysis; N=8 for reproductive hormone test.

Semen from three monkeys was collected and examined. The sperm concentration before scrotal hyperthermia was 66.5 ± 13.0 × 10^6^/ml. The concentration decreased obviously from day 15 after hyperthermia, reaching 0.5 ± 0.3 × 10^6^/ml, approached 0 at days 30 and 45 (both *p* < 0.01 when compared with baseline levels), and recovered to baseline at day 60 (81.0 ± 20.2 × 10^6^/ml). Progressive sperm motility also showed an obvious decrease after hyperthermia and had recovered to baseline at day 60 after hyperthermia. This parameter was not analyzed at days 30 and 45 due to the extremely low sperm concentration. No obvious change was observed in total sperm motility among these observation time points, as shown in Figure 1A-1C. The serum FSH, LH, and TT levels are presented in Figure 1D-1F. No significant differences were observed in the levels before and after heat treatment.

### Testicular histology

**Figure 2:**
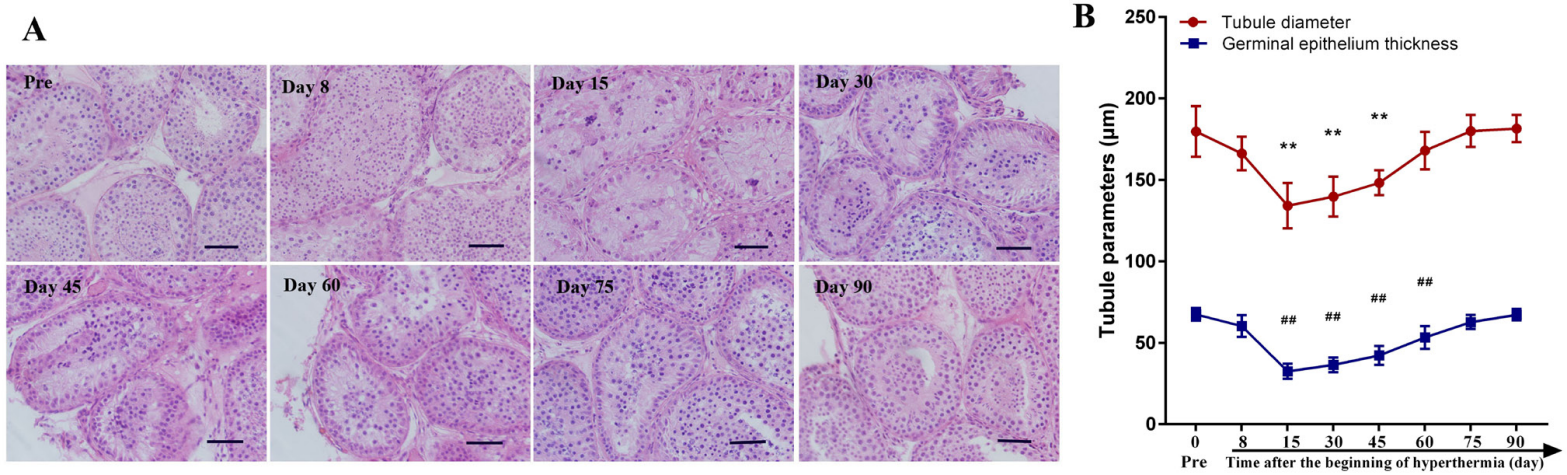
Testicular histology before scrotal hyperthermia and at different time points after hyperthermia **(A)** representative pictures of testicular morphology; **(B)** quantitative analysis of the diameter of seminiferous tubules and the thickness of germinal epithelium. **, *p*<0.01 when compared to baseline, for the diameter of seminiferous tubules; ^##^, *p*<0.01 when compared to baseline, for the thickness of germinal epithelium. Scale bar=50 μm. For each time point, n=4.

Testicular histology was examined after HE staining. The results are presented in Figure 2. The structure of seminiferous epithelium was disrupted at day 8 after hyperthermia with some germ cell sloughing from Sertoli cells. There were also some mature spermatozoa in the lumen during this period, whereas no obvious change was observed in the interstitial space. At days 15 and 30 after treatment, most germ cells had been lost from the seminiferous epithelium, and no obvious change was seen in the Sertoli cells. The structure of the seminiferous epithelium started to recover between days 45 and 75 and had completely recovered to normal by day 90. We analyzed the diameter of seminiferous tubules and the thickness of germinal epithelium, the results showed that the tubule diameter decreased significantly at day 15, 30 and 45 (all *p*<0.01) and tended to recover at day 60. The thickness of seminiferous tubules decreased significantly at day 15, 30, 45 and 60 (all *p*<0.01), and totally recovered to baseline at day 75.

### Identification and quantification of differentially expressed proteins

In total, 5319 proteins were detected by proteome analysis, 3769 of which were trusted proteins when taking FDR <1% and matched peptides >1 as the standard. After quantitatively comparing the proteome profiles of the testes samples collected at different time points, we found that 101 proteins were expressed differentially before treatment and day 8 after treatment, of which 35 were up-regulated (>1.5-fold change, *p* <0.05) and 66 were down-regulated (<0.67-fold change, *p* < 0.05). There were also 24 proteins that were expressed differentially before treatment and day 60 after treatment, of which 23 were up-regulated and one was down-regulated, as shown in Table 1. Detailed information about the differentially expressed proteins identified at days 8 and 60 is presented in Tables 2 and 3, respectively.

**Table 1:** Identification of differentially expressed proteins in monkey testes after heat treatment

	Day 8/pre-treatment	Day 60/pre-treatment
No. of upregulated proteins	35	23
No. of downregulated proteins	66	1
Total number of differentially expressed proteins	101	24

**Table 2:** List of differentially expressed proteins on monkey testis collected at day 8 post-treatment compared with pre-treatment

Protein	Gene	Peptides	Fold change	p-value
Histone H2B, testis	HIST1H2BA	8	0.28	0.025
PREDICTED: y-box-binding protein 2-like	YBX2	13	0.38	0.000
PREDICTED: KH domain-containing, RNA-binding, signal transduction-associated protein 1-like	KHDRBS1 (Sam68)	5	0.44	0.002
calmegin precursor	CLGN	18	0.46	0.000
PREDICTED: hypothetical protein LOC701574	HIST1H2AG	15	0.48	0.000
PREDICTED: high mobility group protein B2-like	HMGB2	4	0.50	0.001
PC4 and SFRS1-interacting protein isoform 2	PSIP1	7	0.50	0.000
heterogeneous nuclear ribonucleoprotein F	HNRNPF	9	0.50	0.000
RecName: Full=Histone H1t; AltName: Full=Testicular H1 histone	HIST1H1T	9	0.51	0.014
heat shock-related 70 kDa protein 2	HSPA2	59	0.52	0.000
Caltractin isoform 2	CETN1	4	0.52	0.037
acylphosphatase-1 isoform a	ACYP1	3	0.52	0.001
PREDICTED: LOW QUALITY PROTEIN: uncharacterized protein C1orf14-like	SHCBP1L	10	0.53	0.000
L-lactate dehydrogenase C chain	LDHC	16	0.54	0.000
ATP-dependent RNA helicase DDX39	DDX39A	15	0.55	0.001
PREDICTED: heterogeneous nuclear ribonucleoprotein A3-like isoform 3	HNRNPA3	18	0.55	0.000
prostaglandin E synthase 3	PTGES3	7	0.56	0.004
PREDICTED: reticulocalbin-2	RCN2	16	0.56	0.002
heterogeneous nuclear ribonucleoprotein C (C1/C2)	LRRC46	21	0.57	0.000
hypothetical protein EGK_08462	LRRC46	8	0.57	0.002
heat shock protein 90kDa alpha (cytosolic), class A member 1	HSP90AA1	66	0.57	0.000
GLIPR1-like protein 1	GLIPR1L1	12	0.57	0.000
40S ribosomal protein S7	RPS7	4	0.58	0.030
cold-inducible RNA-binding protein	CIRBP	7	0.58	0.002
heterogeneous nuclear ribonucleoproteins A2/B1	HNRNPA2B1	26	0.58	0.000
WD repeat-containing protein 62 isoform 1	WDR62	4	0.58	0.010
calmodulin	CALM1	14	0.59	0.000
serine/arginine-rich splicing factor 2	SRSF2	6	0.59	0.007
small ubiquitin-related modifier 2 isoform a precursor	SUMO2	2	0.59	0.003
hepatoma-derived growth factor-like protein 1	HDGFL1	5	0.60	0.008
Histone H1d	HIST1H1D	7	0.60	0.010
leucine zipper transcription factor-like protein 1	LZTFL1	15	0.60	0.000
putative ATP-dependent RNA helicase DDX4 isoform 1	DDX4	27	0.61	0.000
PREDICTED: ecto-ADP-ribosyltransferase 3-like isoform 2	ART3	9	0.61	0.004
hypothetical protein EGK_16207	TKTL2	15	0.63	0.000
PREDICTED: signal recognition particle 14 kDa protein-like	SRP14	2	0.63	0.030
uncharacterized protein LOC704570	C6orf211	4	0.63	0.002
testis-expressed sequence 30 protein	TEX30	2	0.63	0.011
hypothetical protein EGK_02694	BANF2	1	0.64	0.017
hypothetical protein EGK_10689	TEX101	6	0.64	0.031
hypothetical protein EGK_04360	PIWIL1	18	0.64	0.000
sperm surface protein Sp17	SPA17	6	0.64	0.048
heterogeneous nuclear ribonucleoprotein K	HNRNPK	29	0.64	0.000
14-3-3 protein epsilon	YWHAE	26	0.64	0.000
60S ribosomal protein L23	RPL23	6	0.64	0.012
PREDICTED: ELAV-like protein 1 isoform 4	ELAVL1	11	0.64	0.004
hypothetical protein EGK_04675, partial	PPIL3	2	0.64	0.019
Aly/REF export factor	ALYREF	7	0.65	0.030
hypothetical protein EGK_03075	ST13	11	0.65	0.001
transformer-2 protein homolog beta	TRA2B	5	0.65	0.024
histone H2A.x	H2AFX	8	0.65	0.001
DAZ-associated protein 1 isoform b	DAZAP1	9	0.65	0.000
hypothetical protein EGK_04363	RAN	6	0.66	0.010
L-lactate dehydrogenase A-like 6B	LDHAL6B	9	0.66	0.047
T-complex protein 1 subunit zeta-2, partial	CCT6B	21	0.66	0.000
ruvB-like 1	RUVBL1	13	0.66	0.001
peroxiredoxin-5, mitochondrial isoform a precursor	PRDX5	9	0.66	0.009
heat shock 70 kDa protein 4L	HSPA4L	50	0.66	0.000
ATPase inhibitor, mitochondrial	ATPIF1	2	0.66	0.009
dr1-associated corepressor	DRAP1	1	0.66	0.011
peptidylprolyl isomerase D	PPID	19	0.67	0.000
hypothetical protein LOC340277 isoform 1	FAM221A	4	0.67	0.005
glutathione S-transferase M3	GSTM3	31	0.67	0.000
X-ray repair cross-complementing protein 6	XRCC6	25	0.67	0.000
hypothetical protein EGK_12573	44M2.3	7	0.67	0.006
Ubiquilin-3	UBQLN3	2	0.67	0.042
epoxide hydrolase 1	EPHX1	13	1.50	0.000
tubulointerstitial nephritis antigen-like isoform 1 precursor	TINAGL1	7	1.50	0.028
PREDICTED: complement component C9	C9	6	1.51	0.000
RAC-alpha serine/threonine-protein kinase	AKT1	2	1.51	0.048
guanine nucleotide-binding protein G(q) subunit alpha	GNAQ	4	1.53	0.046
dipeptidase 1 precursor	DPEP1	9	1.54	0.028
tropomyosin beta chain isoform 2	TPM2	20	1.56	0.000
thioredoxin	TXN	6	1.57	0.021
beta-hexosaminidase subunit alpha precursor	HEXA	4	1.59	0.049
Plectin	PLEC	107	1.60	0.012
N-acylethanolamine-hydrolyzing acid amidase isoform 1 precursor	NAAA	4	1.60	0.032
decorin isoform a preproprotein	DCN	13	1.61	0.001
PREDICTED: tropomyosin alpha-4 chain isoform 9	TPM1	26	1.61	0.003
immunoglobulin heavy chain	IGHG1	20	1.61	0.000
Olfactomedin-like protein 1	OLFML1	8	1.62	0.001
PREDICTED: annexin A1 isoform 4	ANXA1	18	1.66	0.000
plastin-2	LCP1	7	1.67	0.002
adenosine kinase isoform b	ADK	6	1.69	0.036
keratin, type II cytoskeletal 8	KRT8	13	1.71	0.002
Angiotensin-converting enzyme 2	ACE2	10	1.72	0.006
epidermal retinol dehydrogenase 2	SDR16C5	3	1.74	0.042
PREDICTED: liver carboxylesterase 1-like	CES1	3	1.77	0.029
PREDICTED: serpin B6 isoform 7	SERPINB6	2	1.83	0.019
macrophage-capping protein	CAPG	7	1.84	0.017
hypothetical protein EGK_08282, partial	MFAP4	7	1.89	0.003
calponin-1	CNN1	10	2.01	0.000
hypothetical protein EGK_10576	LGALS7	6	2.02	0.000
PREDICTED: SID1 transmembrane family member 2 isoform 2	SIDT2	17	2.06	0.000
prolargin precursor	PRELP	13	2.14	0.000
hexokinase-2	HK2	6	2.17	0.012
mimecan precursor	OGN	10	2.21	0.000
lumican precursor	LUM	18	2.35	0.000
PREDICTED: collagen alpha-1(XII) chain-like	COL12A1	2	2.64	0.013
fibromodulin precursor	FMOD	3	2.81	0.023
biglycan preproprotein	BGN	15	3.22	0.000

**Table 3:** List of differentially expressed proteins on monkey testis collected at day 60 post-treatment compared with pre-treatment

Protein	Gene	Peptides	Fold change	p-value
Histone H2B, testis	HIST1H2BA	8	0.60	0.021
PREDICTED: 60S ribosomal protein L18a-like isoform 2	RPL18A	2	1.51	0.038
hypothetical protein EGK_18625	IGHM	5	1.51	0.008
decorin isoform a preproprotein	DCN	13	1.52	0.000
keratin, type II cytoskeletal 8	KRT8	13	1.52	0.000
Olfactomedin-like protein 1	OLFML1	8	1.53	0.003
hypothetical protein EGK_17543	ANXA2	27	1.53	0.000
galectin-1	LGALS1	10	1.56	0.000
hypothetical protein EGK_10576	LGALS7	6	1.58	0.001
cochlin precursor	COCH	2	1.59	0.022
thioredoxin	TXN	6	1.59	0.004
hemoglobin subunit beta	HBB	27	1.59	0.000
Coagulation factor XIII A chain	F13A1	6	1.63	0.006
PREDICTED: annexin A1 isoform 4	ANXA1	18	1.64	0.001
hypothetical protein EGK_01534	APOA2	5	1.64	0.004
Putative thymosin beta-4-like protein 1, partial	TMSB4X	2	1.64	0.015
carbonic anhydrase 2	CA2	9	1.68	0.000
hypothetical protein EGK_04859	COL4A3	4	1.68	0.033
prolargin precursor	PRELP	13	1.75	0.008
lumican precursor	LUM	18	1.97	0.000
protein S100-A6	S100A6	4	2.05	0.015
immunoglobulin heavy chain	IGHG1	20	2.12	0.000
biglycan preproprotein	BGN	15	2.22	0.000
hypothetical protein EGK_08282, partial	MFAP4	7	2.38	0.000

### Gene ontology of differentially expressed proteins

**Figure 3:**
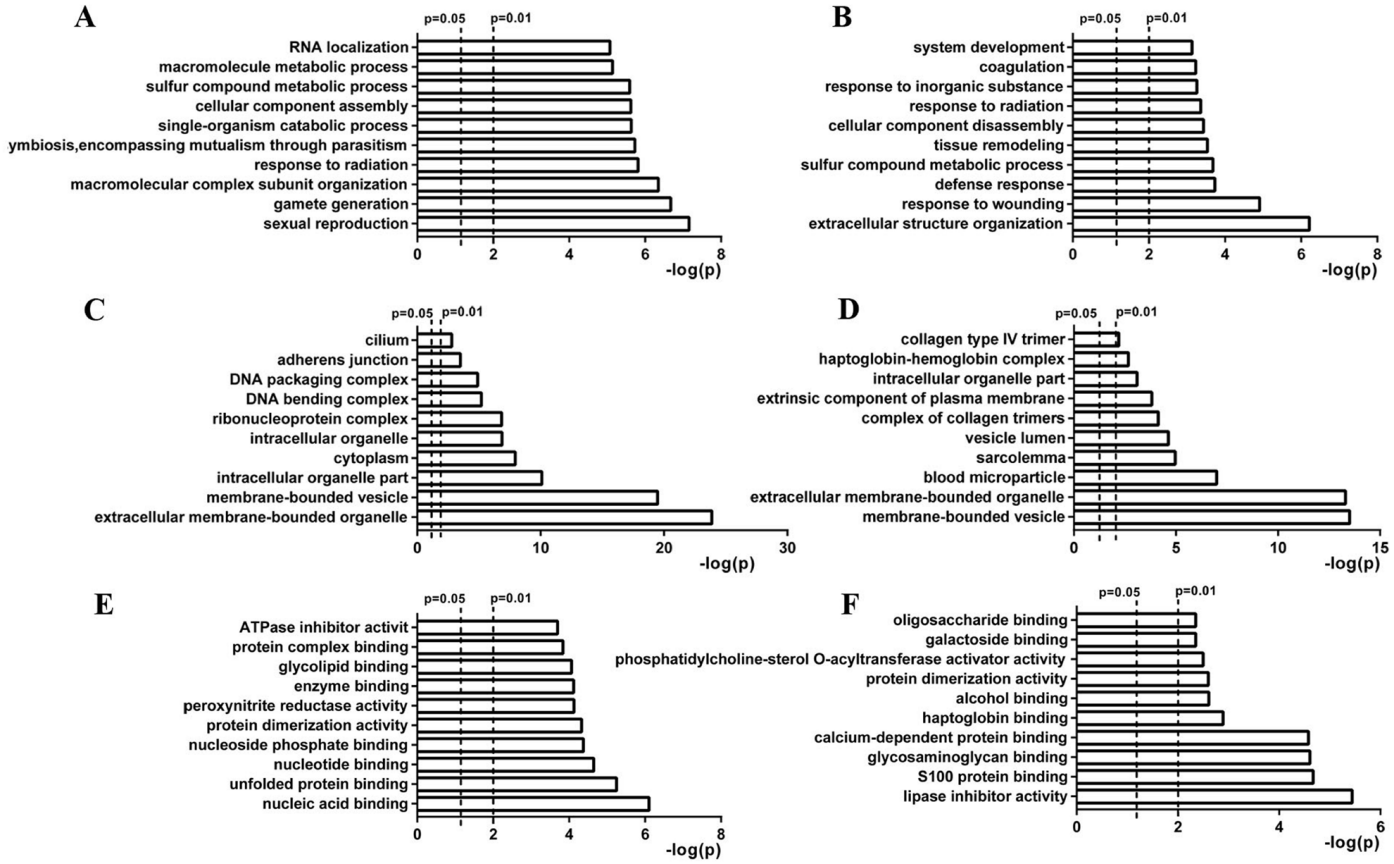
Gene ontology of differentially expressed proteins identified at days 8 and 60 after scrotal hyperthermia Biological process (BP), cell component (CC), and molecular function (MF) are three basic information units of GO analysis. **(A** and **B)** show the top 10 significant BPs enriched for differentially expressed proteins identified at days 8 and 60, respectively. **(C** and **D)** show the top 10 significant CCs enriched for differentially expressed proteins identified at days 8 and 60, respectively. **(E** and **F)** show the top 10 significant MFs enriched for differentially expressed proteins identified at days 8 and 60, respectively.

Gene ontology (GO) analysis was conducted to understand the functional basis of the differentially expressed proteins identified by iTRAQ. Enrichment analysis using the hypergeometric test is used to test whether a GO term is statistically enriched for a given set of genes. The biological process, cell component and molecular function data was the basic information of GO analysis. A total of 113 and 88 biological processes were enriched (*p* <0.05) for the differentially expressed proteins of day 0 vs day 8 and day 0 vs day 60, respectively. The top 10 enriched processes are shown in Figure 3A and 3B. Most of the proteins that differed between day 0 and day 8 were those primarily involved in sexual reproduction, fertilization, and gamete generation. The proteins that differed between day 0 and day 60 were mainly those involved in sexual reproduction, gamete generation, and macromolecular complex subunits. There were 23 and 19 cell components enriched (*p* <0.05) for the differentially expressed proteins of day 0 vs day 8 and day 0 vs day 60, respectively. The top 10 enriched processes are shown in Figure 3C and 3D. In the molecular function analysis, 30 and 19 processes were enriched for the differentially expressed proteins of day 0 vs day 8 and day 0 vs day 60, respectively. The top 10 enriched processes are shown in Figure 3E and 3F.

### KEGG pathway and protein–protein interaction analysis

Kyoto Encyclopedia of Genes and Genomes (KEGG) pathway analysis was conducted to enrich the potential pathways of the identified proteins with differential expression. A total of 14 and 5 pathways (*p* <0.05) were enriched for the differentially expressed proteins of day 0 vs day 8 and day 0 vs day 60, respectively, as shown in Tables 4 and 5. The spliceosome was the most significant KEGG pathway for the altered proteins of day 0 vs day 8, with seven proteins (HNRNPA3, HSPA2, TRA2B, ALYREF, HNRNPC, SRSF2, and HNRNPK) involved. Protein–protein interaction (PPI) analysis was performed using the STRING (Search Tool for the Retrieval of Interacting Genes/Proteins) database. Supplementary Figure 1 show the PPI network combined with KEGG pathways for the differentially expressed proteins of day 0 vs day 8 and day 0 vs day 60, respectively.

**Table 4:** KEGG pathways enriched for altered proteins identified from day 8 testis tissues compared with pre-treatment

Pathway Name	Pathway ID	Genes	p-value
Spliceosome	hsa03040	HNRNPA3, HSPA2, TRA2B, ALYREF, HNRNPC, SRSF2, HNRNPK	3.36E-05
Estrogen signaling pathway	hsa04915	GNAQ, HSP90AA1, HSPA2, AKT1, CALM1	6.68E-04
Pyruvate metabolism	hsa00620	LDHC, ACYP1, LDHAL6B	2.98E-03
Adrenergic signaling in cardiomyocytes	hsa04261	GNAQ, AKT1, TPM1, TPM2, CALM1	3.91E-03
Glycolysis / Gluconeogenesis	hsa00010	LDHC, LDHAL6B, HK2	1.13E-02
Systemic lupus erythematosus	hsa05322	HIST1H2BA, H2AFX, HIST1H2AG, C9	1.63E-02
Propanoate metabolism	hsa00640	LDHC, LDHAL6B	2.15E-02
Cysteine and methionine metabolism	hsa00270	LDHC, LDHAL6B	2.82E-02
Protein processing in endoplasmic reticulum	hsa04141	HSP90AA1, HSPA2, HSPA4L, UBQLN3	3.08E-02
Butirosin and neomycin biosynthesis	hsa00524	HK2	3.51E-02
Alcoholism	hsa05034	HIST1H2BA, H2AFX, HIST1H2AG CALM1	3.83E-02
Carbohydrate digestion and absorption	hsa04973	AKT1, HK2	4.04E-02
Amoebiasis	hsa05146	GNAQ, C9, SERPINB6	4.20E-02
Amino sugar and nucleotide sugar metabolism	hsa00520	HEXA, HK2	4.37E-02

**Table 5:** KEGG pathways enriched for altered proteins identified from day 60 testis tissues compared with pre-treatment

Pathway Name	Pathway ID	Genes	p-value
Nitrogen metabolism	hsa00910	CA2	2.49E-02
Proximal tubule bicarbonate reclamation	hsa04964	CA2	3.36E-02
Collecting duct acid secretion	hsa04966	CA2	3.93E-02
Proteoglycans in cancer	hsa05205	LUM, DCN	4.18E-02
African trypanosomiasis	hsa05143	HBB	4.93E-02

### Validation of iTRAQ data for selected candidate proteins

**Figure 4:**
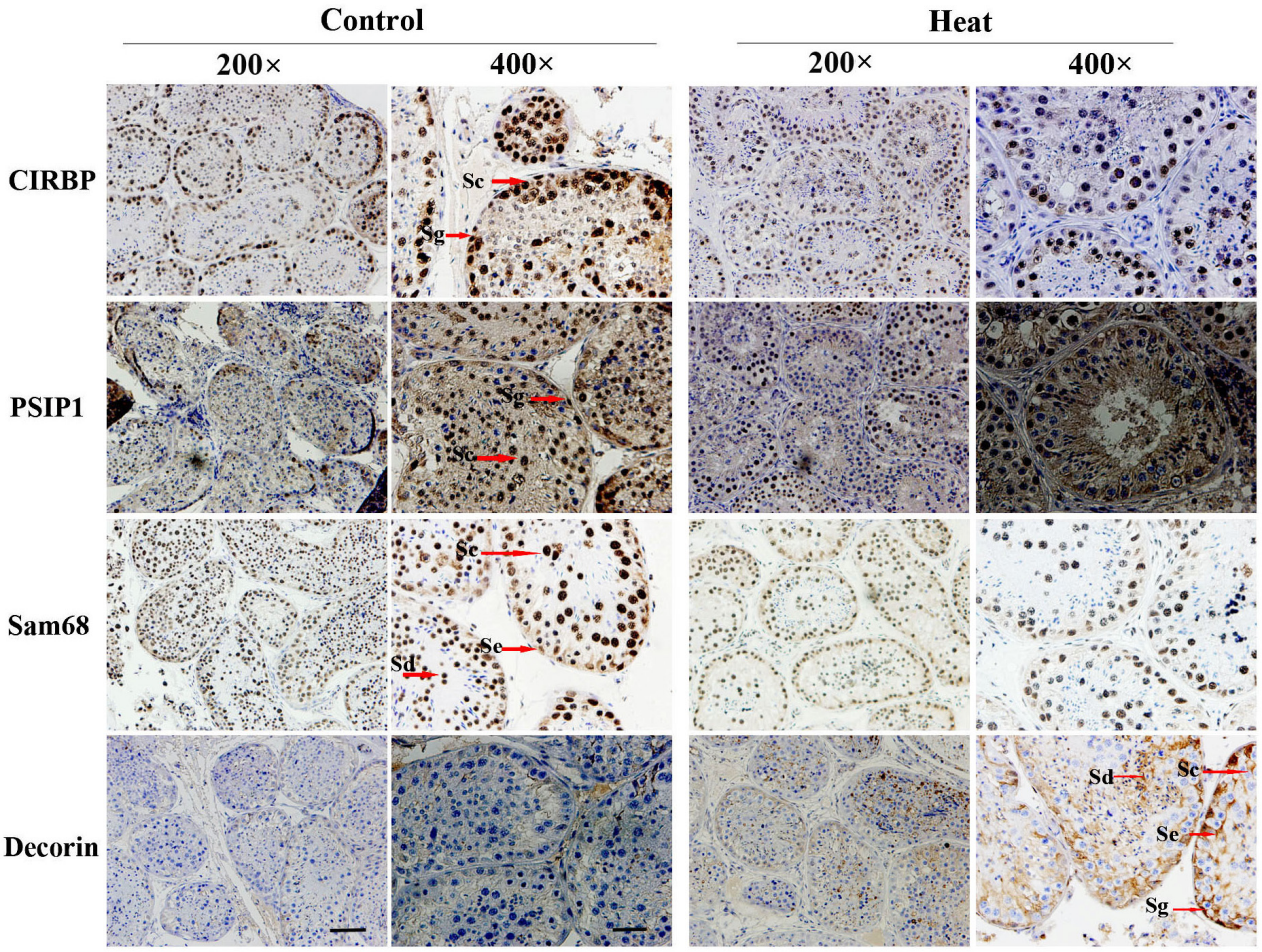
Immunohistochemical staining of CIRBP, PSIP1, Sam68, and Decorin on monkey testis Two representative pictures (200× and 400×) are shown for each marker. Sc, spermatocyte; Sg, spermatogonia; Se, Sertoli cells; Sd, spermatid; Pb, peritubular cell. For 200× images, scale bar=50 μm; for 400× images, scale bar=25 μm.

**Figure 5:**
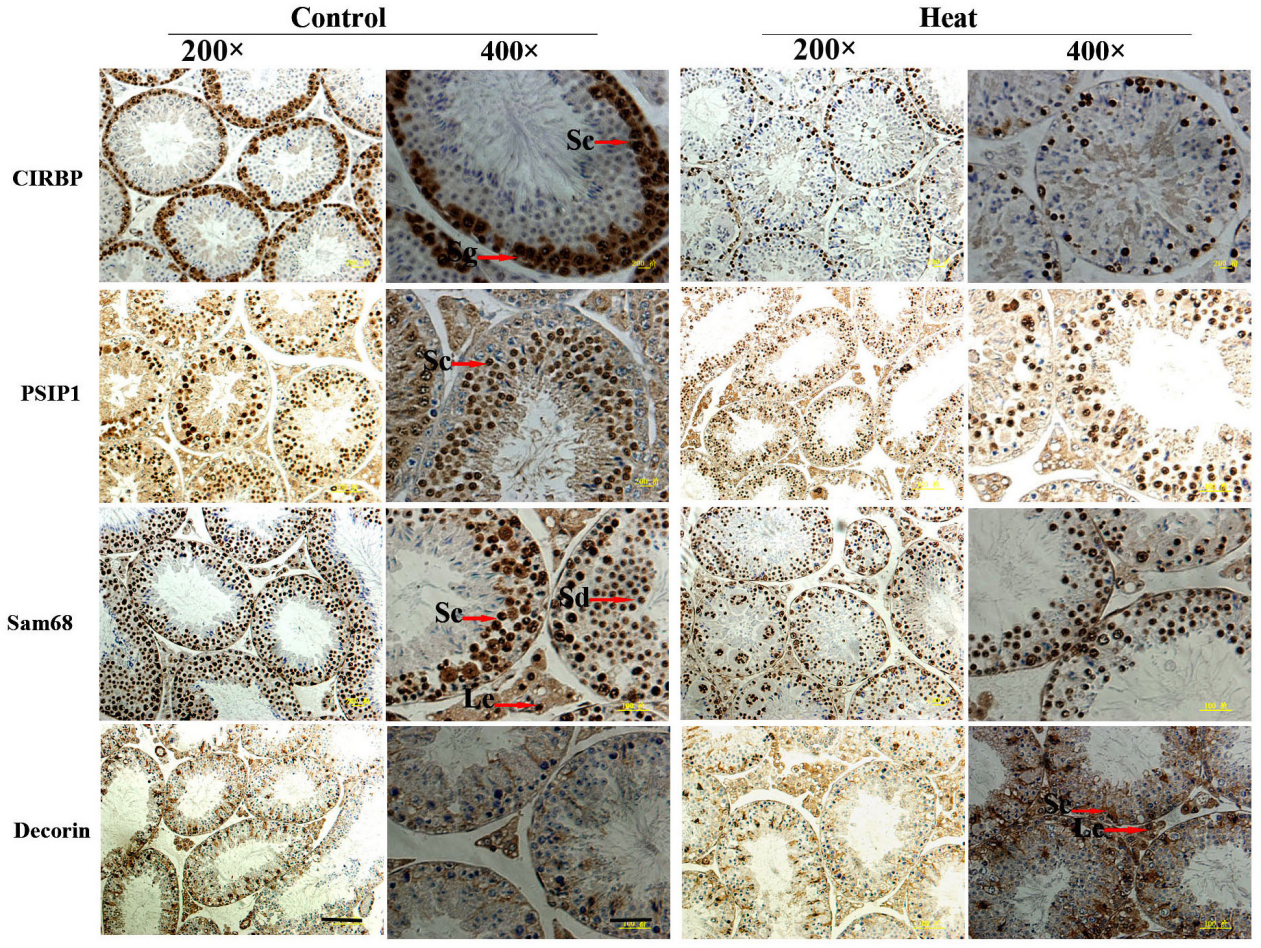
Immunohistochemical staining of CIRBP, PSIP1, Sam68, and Decorin on mouse testis Two representative pictures (100× and 400×) are shown for each marker. Sc, spermatocyte; Sg, spermatogonia; Se, Sertoli cells; Sd, spermatid; Le, Leydig cell; Pb, peritubular cell. For 200× images, scale bar=50 μm; for 400× images, scale bar=25 μm

The results of immunohistochemical analysis showed that PSIP1, CIRBP, Sam68, and decorin were expressed in both monkey and mouse testes, as shown in Figures 4 and 5. In untreated monkey and mouse testes, a high nuclear expression of CIRBP was noted in the germ cells, mainly localized in spermatocytes and spermatogonia, without immunostaining in the Leydig cells or Sertoli cells. PSIP1 was also expressed in the nuclei of the germ cells and was mainly localized in spermatocytes and spermatids, with little or no immunostaining in the spermatogonia. Similarly, Sam68 was also highly expressed in the nuclei of spermatocytes and spermatids, and immunostaining was also detected in the nuclei of Leydig cells. All three proteins were down-regulated after scrotal hyperthermia without any changes in the cellular localization. The expression level of decorin was very low in normal testes, but a higher expression profile was observed after heat treatment, when it was widely expressed in the cytoplasm of spermatocytes, spermatids, and Sertoli cells, with little staining in the spermatogonia in the monkey testes. However, the pattern of expression in the mouse testes was different from that in monkey testes, with some expression in the Leydig cells and Sertoli cells but little expression in the germ cells.

**Figure 6:**
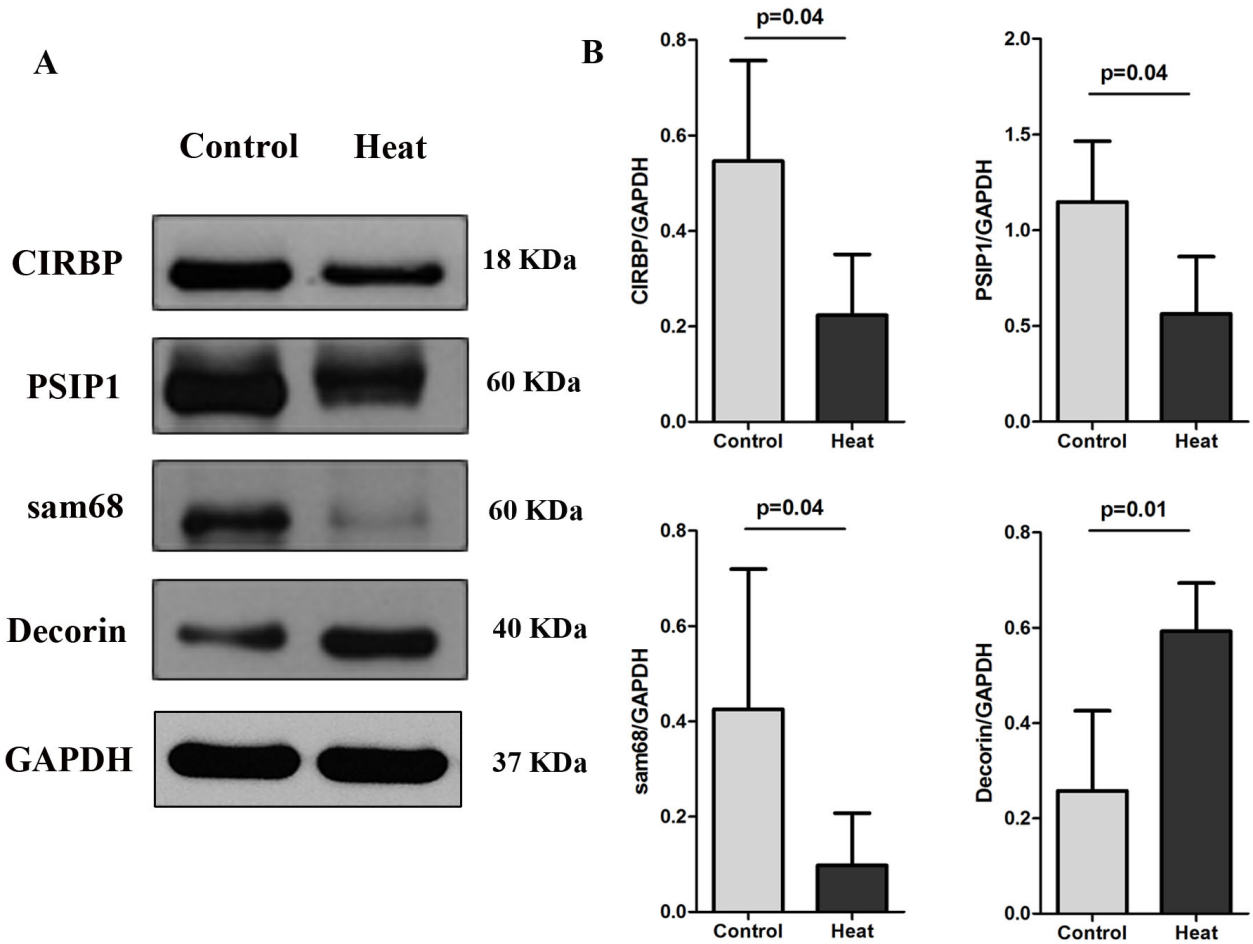
Western blotting results of CIRBP, PSIP1, Sam68, and Decorin on monkey testis analyzed before scrotal hyperthermia and at day 8 after the beginning of hyperthermia For each time point, n=4.

Western blotting analysis in the monkey testis was consistent with the iTRAQ results and immunohistochemical analysis. The relative expression levels of CIRBP, PSIP1, and Sam68 were significantly lower at day 8 after treatment than before treatment (*p* = 0.039, 0.037, and 0.043, respectively). Decorin was significantly up-regulated after heat treatment (*p* = 0.014), as shown in Figure 6.

## DISCUSSION

To our knowledge, this is the first iTRAQ-based proteomic analysis on heat-induced spermatogenic impairment in non-human primates. We attempted to delineate the early testicular cell response to heat treatment and to elucidate the underlying molecular mechanism involved in heat-induced suppression of spermatogenesis.

Similar to our earlier study in humans [7], the suppression of spermatogenesis induced by heat stress was obvious and completely reversible in this study. The sperm concentrations of all three monkeys decreased obviously after heat treatment and exhibited severe oligozoospermia at days 30 and 45 after the first treatment. One of the monkeys even had azoospermia. This was consistent with another study in which monkeys were treated with the same intensity and frequency of scrotal hyperthermia [16]. The serum FSH, LH, and testosterone levels did not change throughout the experiment, indicating that circulating reproductive hormone levels were not affected by the transient heat stress. Our result is consistent with a study published by Lue et al. [17], in which monkeys’ testicles were exposed to heat (43°C for 30 min) for 2 consecutive days, which found that testosterone level was not changed after treatment. However, Hou et al. [18] carried out a study in which rats were exposed to a 40°C environment (whole body) for 2 h per day for 7 consecutive days and found that the serum testosterone concentration significantly decreased after treatment, perhaps as a result of damage to the hypothalamus. The different results may be due to differences in heat treatment strategies, duration, and species.

In this study, a total of 101 and 24 proteins were found to be significantly differentially expressed at days 8 and 60, respectively, when compared to baseline. KEGG pathway analysis enriched 14 and 5 potential pathways at days 8 and 60, respectively. These pathways may be critical in the process of heat-induced spermatogenic impairment and the recovery process. Zhu et al. [11] also investigated differentially expressed proteins at the early and recovery stages of heat induced testicular damage in men, by using two-dimensional SDS-PAGE and mass spectrometry (2DE-MS), and found 32 and 26 differentially expressed proteins at week 2 and week 9 after heat treatment, when taking a 1.2-fold change as a standard. However, our study identified more differentially expressed proteins, especially at the early stage of damage. It is worth noting that the standard in our study was 1.5-fold change. It was difficult to compare the enriched pathways between that study and ours, since the bioinformatics analysis between these two studies were different. Nevertheless, both studies enriched spliceosome as one the most significant pathways at the early stage of spermatogenic impairment. In that study, the investigators found that heat stress–induced alteration of heterogeneous nuclear ribonucleoprotein (hnRNP) expression was closely related to germ cell apoptosis [11]. In this study, we found that spliceosome was associated with seven differentially expressed proteins (HNRNPA3, HSPA2, TRA2B, ALYREF, HNRNPC, SRSF2, HNRNPK). The spliceosome is a conserved, very large complex that consists of five small nuclear ribonucleoprotein (snRNP) complexes (U1, U2, U4, U5, and U6) and approximately 150 proteins [19]. The spliceosome removes introns from a transcribed pre-mRNA and thus plays an important role in gene expression [20, 21]. Studies have shown that the spliceosome is also critical in normal spermatogenesis. As a component of the spliceosome, U2A loss in germ cells of *Drosophila* testes caused insufficient splicing of mRNAs required for the transition of germ cells from proliferation to differentiation, resulting in the accumulation of mitotic spermatogonia that failed to differentiate into spermatocytes and mature sperm [22]. Alikhani et al. [23] carried out a proteomic analysis on testicular tissue specimens and found that RNA splicing was one of the most significantly altered biological processes in patients with Sertoli cell–only syndrome. Heat exposure altered the expression of some protein components of the spliceosome and resulted in dysfunction of RNA splicing in its target pre-mRNAs. This process may be critical in heat-induced germ cell apoptosis.

Our proteomic analysis also revealed some protein targets and pathways which played important roles in germ cell apoptosis. Heat shock-related 70 kDa protein 2 (HSPA2) and SHC binding and spindle associated 1 like (SHCBP1L) were identified proteins with 0.52 and 0.53-fold change of expression, respectively in our proteomic analysis. Studies showed that SHCBP1L binded to HSPA2, and maintained stability of the spindle [24](a). Both HSPA2 and SHCBP1L gene defencicy led to germ cell apoptosis [24, 25]. Protein processing in endoplasmic reticulum was enriched as another significant pathway, indicating that heat stress affected the function of endoplasmic reticulum. It is known that endoplasmic reticulum (ER) dysfunction would lead to ER stress, which is closely related to cell apoptosis [26, 27]. Some other metabolism-related pathways like glucolysis/gluconeogenesis, cysteine and methionine metabolism as well as pyruvate metabolism were also enriched as significant pathways during the early stage of testicular damage. These altered metabolism status may also involved in the heat stress-induced spermatogenic impairment.

PSIP1, CIRBP, Sam68, and decorin were selected for validation for the iTRAQ data. Both immunohistochemical analysis and western blotting results were consistent with the iTRAQ results, indicating the reliability of the sample pooling strategy in the proteomic approach. CIRBP is an RNA-binding protein that participates in forming ribonucleoprotein complexes [28]. Nishiyama et al. [29] found the that CIRBP expression was down-regulated at elevated temperatures in germ cells of male mice and humans. Masuda et al. [30] investigated the function of CIRBP using a gene knockout model and showed that a *Cirbp* gene defect obviously suppressed the proliferation of undifferentiated spermatogonia. PSIP1, another RNA binding protein, was expressed in spermatocytes and spermatids. It has been reported that PSIP1 is closely involved in spermatomiosis [31]. Sam68 has also been found to be closely related to spermatogenesis and plays an important role in regulating the expression of meiosis-related genes [32, 33]. A lack of Sam68 was found to cause decreased production of spermatozoa, which also displayed dramatic motility defects and were unable to fertilize eggs [33]. Li et al. [34] found a Sam68 expression deficiency in human testes, with maturation arrest at the spermatocyte stage and Sertoli cell–only syndrome in comparison with normal spermatogenesis. Furthermore, decreased expression of Sam68 suppressed germ cell proliferation and induced apoptosis in transfected GC-2spd cells. Decorin in the testis is mainly produced by myofibroblastic peritubular cells in the walls of seminiferous tubules [35, 36]. Adam et al. [35] reported an increased concentration of testicular decorin in infertile men, and the increase in decorin may consequently imbalance the paracrine signaling pathways in human testes, which has also been demonstrated in mice and monkeys [36]. It is worthy of note that only four of the differentially expressed proteins were selected for validation of the iTRAQ results; other proteins may also be very important molecular targets involved in heat-induced spermatogenic impairment.

In summary, this study investigated the testicular proteomic profiles of monkeys exposed to transient scrotal hyperthermia. A total of 101 and 24 proteins were found to be differentially expressed at the early and recovery stages of testicular damage respectively, when compared to pretreatment. Most of the identified and differentially regulated proteins at the early stage of damage can be divided into the following categories according to their molecular functions: nucleotide binding, and nucleoside phosphate binding, unfolded protein binding, nucleotide binding, and nucleoside phosphate binding. The most significant pathway for these differentially expressed proteins at the early stage of damage was the spliceosome, with seven proteins involved. CIRBP, PSIP1, Sam68, and decorin were validated and found to be consistent with the proteomic data, indicating the reliability of the proteomic profiles identified in this study. We suggest that the proteins identified in this study play important roles in heat-induced spermatogenic impairment. Further study is required to clarify the roles of these molecular targets in the pathogenesis of heat-induced male infertility.

## MATERIALS AND METHODS

### Animals and study design

This study was approved by the Animal Care and Use Committee at Fujian provincial Population and Family Planning Research Institute. Eight male adult (8 to 10 years old) rhesus monkeys were obtained and housed at the Fuzhou Primate Research Center, Family Planning Research Institute of Fujian Province. The monkeys were housed in a standard animal facility under controlled temperature (22°C) and photoperiod (12 h of light and 12 h of darkness) with free access to water and food. All monkeys were subjected to scrotal hyperthermia at 43°C for 30 min once daily for 6 consecutive days, as previously described [16]. In brief, after monkeys were anesthetized with an intramuscular injection of ketamine (5 to 10 mg/kg body weight), their scrota were immersed in a thermostatically controlled water bath at 43°C for 30 min. After heat treatment, the animals were dried, examined for any injury to the testes, and returned to their cages. No injuries to the monkeys’ scrotal skin were found during this process.

Semen samples, blood, and testes biopsy tissues were collected 2 weeks before scrotal hyperthermia and at days 8, 15, 30, 45, 60, 75, and 90 after the beginning of hyperthermia. To avoid damage from frequent biopsies to the testes, the eight monkeys were randomly divided into two groups, four monkeys in each group. Biopsies were taken alternately from the two groups and also from testes on alternate sides. Thus, for each animal, two biopsies were taken from the left testis and another two from the right testis.

### Semen collection and analysis

Semen samples were collected using an electronic stimulator provided by the Fuzhou Primate Research Center (self-made). A stimulus was produced when the alternating current sine wave pulse went through a ring electrode. After the monkeys were anesthetized with an intramuscular injection of ketamine (5 mg/kg body weight), the stimulator was lubricated and inserted into the anus to about 9 cm. The stimulator was then electrified every 5 seconds. The penis became erect to ejaculate as the voltage increased from 1.3 V→2.1 V→3.6 V→7.2 V→8.6 V. Each ejaculate included both fluid and coagulum fractions. The sperm concentration and motility were determined from the fluid fraction using a hemocytometer and expressed as ×10^6^/ml. Both progressive sperm motility and total sperm motility were examined. Semen samples from three monkeys were collected and analyzed at each time point.

### Blood collection and hormone assay

Blood samples were collected from an arm vein while the monkeys were briefly restrained, and serum was separated and stored at −80°C for subsequent assays of follicle stimulating hormone (FSH), luteinizing hormone (LH), and total testosterone (TT) using enzyme-linked immunosorbent assay with kits provided by Cusabio Biotech (Wuhan, China). The lower limits of quantitation for FSH, LH, and TT were 0.2 mIU/ml, 0.5 mIU/ml, and 0.05 ng/ml, respectively. The intra-assay and inter-assay coefficients of variation for these three parameters were all less than 15%.

### Testicular biopsy and morphological observation

Open testicular biopsies were performed under aseptic conditions, after the monkeys had been anesthetized with ketamine (10 mg/kg body weight). Testicular tissues from each monkey were divided into three samples. One sample was fixed in Bouin’s solution immediately for morphological observation, and the remainder was stored at −80°C for gene and protein analysis. Morphological evaluation was performed with hematoxylin and eosin (HE) staining. Briefly, sections from paraffin-embedded tissues were dewaxed in xylene, hydrated through a graded series of decreasing concentrations of alcohol, and stained with hematoxylin and eosin separately. After washing, the slides were dehydrated through a series of graded alcohols and xylene and mounted with neutral resin. Testicular histology was evaluated by quantifying the diameter of seminiferous tubules and the thickness of germinal epithelium as described in other studies [37, 38] Briefly, 30 transverse sections of seminiferous tubules from each slide were randomly selected. Diameter of each tubule was measured across the minor axe of its profile with an ocular micrometer calibrated by a stage micrometer. Thickness of germinal epithelium was quantified by measuring the distance from the lumen to the basement membrane. The average value from those 30 tubules were regarded as the value of one specific animal, 4 animals were analyzed at each time point.

### iTRAQ analysis

#### Protein extraction and preparation

Testicular tissues collected before scrotal hyperthermia (day 0) and at days 8, 30, and 60 after hyperthermia were used for iTRAQ analysis. Four tissue samples (30 mg) from each time point were pooled separately. The pooled samples were ground to powder with liquid nitrogen and dissolved in a lysis solution (9 mol/L urea, 4% CHAPS, 1% IPG buffer, 1% DTT) at 30°C for 1 h. Following two centrifugations at 15,000*g* for 15 min at room temperature, the supernatant was collected and the concentrations of the protein extracts were determined by the Bradford method [39]. A sample of 100 μg protein from each pooled sample was used for iTRAQ labeling according to the manufacturer’s protocol (ABI System, USA). Briefly, five volumes of cold acetone were added to each sample tube, vortexed and then held at −20°C for 1 h. After centrifugation at 12,000 rpm for 15 min at 4°C, the deposit was collected and dried in a vacuum freeze dryer. Then 50 μl dissolution buffer and 4 μl reducing reagent were added to the deposit and incubated at 60°C for 1 h. Following incubation with 2 μl cysteine-blocking reagents at room temperature for 10 min, the solution was cleaned with a 10-KDa ultrafiltration tube and centrifuged at 12,000 rpm for 20 min. Next, 100 μl dissolution buffer was added to wash the proteins at 12,000 rpm three times for 15 min. After washing, 50 μl trypsin (50 ng/μl) was added and incubated at 37°C for 12 h to digest each sample. After centrifugation at 12,000 rpm for 20 min to collect the peptides, the filter unit was transferred to a new collection tube and 50 μl dissolution buffer was added to centrifuge again, and the two filter solutions were combined for protein labeling.

#### Protein labeling and MS analysis

Each iTRAQ reagent was brought to room temperature and spun down to bring the solution to the bottom of the tube before use. Each iTRAQ reagent was dissolved in 150 μl of ethanol, then this reagent was added to 50 μl samples (100 μg peptide) and incubated at room temperature for 2 h, after which 100 μl of Milli-Q water was added to stop the labeling reaction. Then, 1 μl content of each iTRAQ reagent-labeled sample was pooled, cleaned up using a Ziptip, and tested by MALDI-TOF/TOF analysis to ensure the effect of labeling. The samples were pooled, spun, and dried in a vacuum freeze dryer for iTRAQ analysis.

### Strong cation exchange (SCX) analysis

Sample fractionation was performed by strong cation exchange (SCX) chromatography on the Agilent 1200 HPLC System. The HPLC column was from Michrom. The parameters were Poly-SEA 5μ 300 Å 2.0 × 150 mm, flow rate 0.5 ml/min with 215 nm and 280 nm UV detection. In brief, the dry sample was re-suspended with 100 μl SCX buffer A (10 mM formic acid, 20% acetonitrile), fractionated, and collected as 12 fractions. The first fraction was collected from 0 to 5 min, another 10 fractions were collected from 6 to 44 min at intervals of 4 min, and the final fraction was collected from 45 to 50 min. Each fraction was dried in a vacuum freeze dryer for LC-MS/MS analysis.

### Reversed-phase nanoliquid chromatography–tandem MS (RPLC-MS/MS) analysis

Online Nano-RPLC was used on an Eksigent nanoLC-Ultra™ 2D System (AB SCIEX). The samples were re-suspended with Nano-RPLC buffer A (0.1% formic acid, 2% acetonitrile), loaded on a C18 nanoLC trap column (100 μm × 3 cm, C18, 3-μm particle size, 150 Å), and washed with Nano-RPLC buffer A at a flow rate of 2 μl/min for 10 min. A linear LC gradient profile was used to elute peptides from the analytical ChromXP C18 column (75 μm × 15 cm, C18, 3-μm particle size, 120 Å, Eksigent). The gradient began with 5% Nano-RPLC buffer B (0.1% formic acid, 98% acetonitrile) and rose to 35% within 70 min. Data acquisition was performed with a Triple TOF 5600 System (AB SCIEX, USA) fitted with a Nanospray III source (AB SCIEX, USA) and a pulled quartz tip as the emitter (New Objectives, USA). Data were acquired using an ion spray voltage of 2.5 kV, curtain gas of 30 PSI, nebulizer gas of 5 PSI, and an interface heater temperature of 150°C. For information dependent acquisition, survey scans were acquired in 250 ms, and as many as 35 product ion scans were collected if they exceeded a threshold of 150 counts per second (counts/s) with a 2+ to 5+ charge-state. The total cycle time was fixed to 2.5 s. A rolling collision energy setting was applied to all precursor ions for collision-induced dissociation.

### Protein identification and quantification

Data was processed with ProteinPilot v.4.5 (AB SCIEX, USA) and a *Macaca mulatta* database using the Paragon algorithm [40]. Protein identification was performed with the search option “emphasis on biological modifications.” The database search parameters were as follows: the instrument was TripleTOF 5600, iTRAQ 4-plex quantification, cysteine modified with iodoacetamide, biological modifications as the ID focus, trypsin digestion. An automatic decoy database search strategy was used to estimate the false discovery rate (FDR) using PSPEP (Proteomics System Performance Evaluation Pipeline) software, integrated with ProteinPilot. The FDR was calculated as the false-positive matches divided by the total matches. The iTRAQ 4-plex was chosen for protein quantification with unique peptides during the search. A total of 3789 proteins were identified and considered for further analysis. Proteins with corrected average fold changes of >1.50 or <0.67 and *p* <0.05 were considered to be significantly differentially expressed.

### Mice scrotal heat treatment

To validate whether the differentially expressed proteins identified by the iTRAQ analysis showed the same pattern of change in mice before and after heat stress, we induced mice scrotal hyperthermia, as described in another study [41]. Briefly, 10 male adult ICR mice (8 to 9 weeks old) were used; 5 were subjected to scrotal heat treatment at 43°C for 30 min, and the other 5 were controls. All mice were anaesthetized with 1% pentobarbital sodium by intraperitoneal injection (50 mg/kg body weight). In the heat treatment group, the mice scrota were immersed in a thermostatically controlled water bath at 43°C for 30 min. The control mice were left at room temperature. After the heat treatment, the animals were dried and returned to their cages. The mice were sacrificed 12 h after heat treatment. One testis of each mouse was fixed in Bouin’s solution for immunohistochemical examination, and the other testis was frozen in liquid nitrogen for western blot analysis.

### Western blotting

From the differentially expressed proteins, we selected the proteins PC4 and SFRS1 interacting protein 1 (PSIP1), Src-associated substrate in mitosis of 68 kDa (Sam68), cold-inducible RNA-binding protein (CIRBP), and decorin for western blotting to validate their expression levels in the monkey testes before and after hyperthermia. It should be noted that the western blot analysis was based on four individual subjects, not pooled tissue samples. Samples containing 40 μg total proteins were separated by 10% (PSIP1, Sam68, decorin) or 15% (CIRBP) SDS–PAGE. After transfer onto PVDF membranes by electroblotting, the proteins were then probed with the following primary antibodies: CIRBP polyclonal antibody (Abcam, ab106230, 1:1000 dilution), PSIP1 polyclonal antibody (Abcam, ab70641, 1:500 dilution), Sam68 polyclonal antibody (Abcam, ab86239, 1:1000 dilution), decorin polyclonal antibody (Abcam, ab35378, 1:1000 dilution), and GAPDH polyclonal antibody (Abcam, ab37168, 1:10000 dilution). After washing three times with TBST for 5 min, the membranes were incubated with appropriate horseradish peroxidase conjugated goat anti-rabbit (KPL, 074-1506, 1:10000 dilution), rabbit anti-goat (KPL, 14-13-06, 1:10000), or rabbit anti-sheep (Aspen, AS-1114, 1:10000) secondary antibodies. Specific proteins were detected using an ECL kit (Aspen) and exposed to film. The protein expression level was analyzed by AlphaEaseFC software (Alpha Innotech) and the data were normalized using GAPDH. N=4 at each time point.

### Immunohistochemical analysis

Paraffin-embedded sections from the monkey and mouse testis tissue before and after heat stress were used for immunohistochemical analysis. Briefly, after dewaxing and hydration, sections were treated with H_2_O_2_ to quench the endogenous peroxidase activity, blocked using a blocking serum and then incubated overnight at 4°C with primary antibodies to PSIPS (1:100), CIRBP (1:50), Sam68 (1:100), and decorin (1:100). The sections were then incubated with HRP-conjugated secondary antibodies (KPL, 1:400). Immunostaining was developed with a diaminobenzidine kit and counterstained with hematoxylin, and the sections were then dehydrated and mounted.

### Statistical analysis

Sperm parameters, reproductive hormones, and relative protein expression levels are presented as mean ± SD. Sperm parameters and reproductive hormones after hyperthermia were compared with the baseline levels by means of a paired non-parametric Wilcoxon test. Histological parameters (diameter of seminiferous tubules and thickness of germinal epithelium) evaluated at each time points were compared using one-way ANOVA and the LSD test. Relative protein expression levels before and after hyperthermia were compared using the Mann–Whitney U-test. Statistical analysis was performed with SPSS 17.0 software (SPSS Inc., Chicago, IL, USA); a *p* level of less than 0.05 was considered to indicate statistical significance.

## SUPPLEMENTARY MATERIALS FIGURE



## Abbreviations

iTRAQ: isobaric tags for relative and absolute quantification
LC-MS/MS: liquid chromatography–tandem mass spectrometry
FDR: false discovery rate
GO: gene ontology
KEGG: Kyoto Encyclopedia of Genes and Genomes
PSIP1: PC4 and SFRS1 interacting protein 1
Sam68: Src-associated substrate in mitosis of 68 kDa
CIRBP: cold-inducible RNA-binding protein

## References

[1] Ivell R (2007). Lifestyle impact and the biology of the human scrotum. Reprod Biol Endocrinol.

[2] Liu Y, Li X (2010). Molecular basis of cryptorchidism-induced infertility. Sci China Life Sci.

[3] Shiraishi K, Takihara H, Matsuyama H (2010). Elevated scrotal temperature, but not varicocele grade, reflects testicular oxidative stress-mediated apoptosis. World J Urol.

[4] Thonneau P, Ducot B, Bujan L, Mieusset R, Spira A (1997). Effect of male occupational heat exposure on time to pregnancy. Int J Androl.

[5] Hjollund NH, Bonde JP, Jensen TK, Olsen J (2000). The Danish First Pregnancy Planner Study Team. Diurnal scrotal skin temperature and semen quality. Int J Androl.

[6] Rao M, Xia W, Yang J, Hu LX, Hu SF, Lei H, Wu YQ, Zhu CH (2016). Transient scrotal hyperthermia affects human sperm DNA integrity, sperm apoptosis, and sperm protein expression. Andrology.

[7] Rao M, Zhao XL, Yang J, Hu SF, Lei H, Xia W, Zhu CH (2015). Effect of transient scrotal hyperthermia on sperm parameters, seminal plasma biochemical markers, and oxidative stress in men. Asian J Androl.

[8] Hikim AP, Lue Y, Yamamoto CM, Vera Y, Rodriguez S, Yen PH, Soeng K, Wang C, Swerdloff RS (2003). Key apoptotic pathways for heat-induced programmed germ cell death in the testis. Endocrinology.

[9] Vera Y, Diaz-Romero M, Rodriguez S, Lue Y, Wang C, Swerdloff RS, Sinha Hikim AP (2004). Mitochondria-dependent pathway is involved in heat-induced male germ cell death: lessons from mutant mice. Biol Reprod.

[10] Rocha DR, Martins JA, van Tilburg MF, Oliveira RV, Moreno FB, Monteiro-Moreira AC, Moreira RA, Araujo AA, Moura AA (2015). Effect of increased testicular temperature on seminal plasma proteome of the ram. Theriogenology.

[11] Zhu H, Cui Y, Xie J, Chen L, Chen X, Guo X, Zhu Y, Wang X, Tong J, Zhou Z, Jia Y, Lue YH, Hikim AS (2010). Proteomic analysis of testis biopsies in men treated with transient scrotal hyperthermia reveals the potential targets for contraceptive development. Proteomics.

[12] Zhu YF, Cui YG, Guo XJ, Wang L, Bi Y, Hu YQ, Zhao X, Liu Q, Huo R, Lin M, Zhou ZM, Sha JH (2006). Proteomic analysis of effect of hyperthermia on spermatogenesis in adult male mice. J Proteome Res.

[13] Yu S, Cai X, Sun L, Zuo Z, Mipam T, Cao S, Shen L, Ren Z, Chen X, Yang F, Deng J, Ma X, Wang Y (2016). Comparative iTRAQ proteomics revealed proteins associated with spermatogenic arrest of cattleyak. J Proteomics.

[14] Li G, Li M, Liang X, Xiao Z, Zhang P, Shao M, Peng F, Chen Y, Li Y, Chen Z (2017). Identifying DCN and HSPD1 as Potential Biomarkers in Colon Cancer Using 2D-LC-MS/MS Combined with iTRAQ Technology. J Cancer.

[15] Niu D, Sui J, Zhang J, Feng H, Chen WN (2009). iTRAQ-coupled 2-D LC-MS/MS analysis of protein profile associated with HBV-modulated DNA methylation. Proteomics.

[16] Lue YH, Lasley BL, Laughlin LS, Swerdloff RS, Hikim AP, Leung A, Overstreet JW, Wang C (2002). Mild testicular hyperthermia induces profound transitional spermatogenic suppression through increased germ cell apoptosis in adult cynomolgus monkeys (Macaca fascicularis). J Androl.

[17] Lue Y, Wang C, Liu YX, Hikim AP, Zhang XS, Ng CM, Hu ZY, Li YC, Leung A, Swerdloff RS (2006). Transient testicular warming enhances the suppressive effect of testosterone on spermatogenesis in adult cynomolgus monkeys (Macaca fascicularis). J Clin Endocrinol Metab.

[18] Hou Y, Wang X, Lei Z, Ping J, Liu J, Ma Z, Zhang Z, Jia C, Jin M, Li X, Li X, Chen S, Lv Y (2015). Heat-stress-induced metabolic changes and altered male reproductive function. J Proteome Res.

[19] Wahl MC, Will CL, Luhrmann R (2009). The spliceosome: design principles of a dynamic RNP machine. Cell.

[20] Gallego-Paez LM, Bordone MC, Leote AC, Saraiva-Agostinho N, Ascensao-Ferreira M, Barbosa-Morais NL (2017). Alternative splicing: the pledge, the turn, and the prestige : The key role of alternative splicing in human biological systems. Hum Genet.

[21] Gault CM, Martin F, Mei W, Bai F, Black JB, Barbazuk WB, Settles AM (2017). Aberrant splicing in maize rough endosperm3 reveals a conserved role for U12 splicing in eukaryotic multicellular development. Proc Natl Acad Sci U S A.

[22] Wu H, Sun L, Wen Y, Liu Y, Yu J, Mao F, Wang Y, Tong C, Guo X, Hu Z, Sha J, Liu M, Xia L (2016). Major spliceosome defects cause male infertility and are associated with nonobstructive azoospermia in humans. Proc Natl Acad Sci USA.

[23] Alikhani M, Mirzaei M, Sabbaghian M, Parsamatin P, Karamzadeh R, Adib S, Sodeifi N, Gilani MA, Zabet-Moghaddam M, Parker L, Wu Y, Gupta V, Haynes PA (2017). Quantitative proteomic analysis of human testis reveals system-wide molecular and cellular pathways associated with non-obstructive azoospermia. J Proteomics.

[24] Liu M, Shi X, Bi Y, Qi L, Guo X, Wang L, Zhou Z, Sha J (2014). SHCBP1L, a conserved protein in mammals, is predominantly expressed in male germ cells and maintains spindle stability during meiosis in testis. Mol Hum Reprod.

[25] Widlak W, Vydra N, Malusecka E, Dudaladava V, Winiarski B, Scieglinska D, Widlak P (2007). Heat shock transcription factor 1 down-regulates spermatocyte-specific 70 kDa heat shock protein expression prior to the induction of apoptosis in mouse testes. Genes Cells.

[26] Ji YL, Wang Z, Wang H, Zhang C, Zhang Y, Zhao M, Chen YH, Meng XH, Xu DX (2012). Ascorbic acid protects against cadmium-induced endoplasmic reticulum stress and germ cell apoptosis in testes. Reprod Toxicol.

[27] Ji YL, Wang H, Zhang C, Zhang Y, Zhao M, Chen YH, Xu DX (2013). N-acetylcysteine protects against cadmium-induced germ cell apoptosis by inhibiting endoplasmic reticulum stress in testes. Asian J Androl.

[28] Lunde BM, Moore C, Varani G (2007). RNA-binding proteins: modular design for efficient function. Nat Rev Mol Cell Biol.

[29] Nishiyama H, Danno S, Kaneko Y, Itoh K, Yokoi H, Fukumoto M, Okuno H, Millan JL, Matsuda T, Yoshida O, Fujita J (1998). Decreased expression of cold-inducible RNA-binding protein (CIRP) in male germ cells at elevated temperature. Am J Pathol.

[30] Masuda T, Itoh K, Higashitsuji H, Higashitsuji H, Nakazawa N, Sakurai T, Liu Y, Tokuchi H, Fujita T, Zhao Y, Nishiyama H, Tanaka T, Fukumoto M (2012). Cold-inducible RNA-binding protein (Cirp) interacts with Dyrk1b/Mirk and promotes proliferation of immature male germ cells in mice. Proc Natl Acad Sci USA.

[31] Chapman KM, Powell HM, Chaudhary J, Shelton JM, Richardson JA, Richardson TE, Hamra FK (2013). Linking spermatid ribonucleic acid (RNA) binding protein and retrogene diversity to reproductive success. Mol Cell Proteomics.

[32] Paronetto MP, Messina V, Barchi M, Geremia R, Richard S, Sette C (2011). Sam68 marks the transcriptionally active stages of spermatogenesis and modulates alternative splicing in male germ cells. Nucleic Acids Res.

[33] Paronetto MP, Messina V, Bianchi E, Barchi M, Vogel G, Moretti C, Palombi F, Stefanini M, Geremia R, Richard S, Sette C (2009). Sam68 regulates translation of target mRNAs in male germ cells, necessary for mouse spermatogenesis. J Cell Biol.

[34] Li LJ, Zhang FB, Liu SY, Tian YH, Le F, Lou HY, Huang HF, Jin F (2014). Decreased expression of SAM68 in human testes with spermatogenic defects. Fertil Steril.

[35] Adam M, Schwarzer JU, Kohn FM, Strauss L, Poutanen M, Mayerhofer A (2011). Mast cell tryptase stimulates production of decorin by human testicular peritubular cells: possible role of decorin in male infertility by interfering with growth factor signaling. Hum Reprod.

[36] Adam M, Urbanski HF, Garyfallou VT, Welsch U, Kohn FM, Ullrich Schwarzer J, Strauss L, Poutanen M, Mayerhofer A (2012). High levels of the extracellular matrix proteoglycan decorin are associated with inhibition of testicular function. Int J Androl.

[37] Wang C, Cui YG, Wang XH, Jia Y, Sinha Hikim A, Lue YH, Tong JS, Qian LX, Sha JH, Zhou ZM, Hull L, Leung A, Swerdloff RS (2007). transient scrotal hyperthermia and levonorgestrel enhance testosterone-induced spermatogenesis suppression in men through increased germ cell apoptosis. J Clin Endocrinol Metab.

[38] Bakhtiary Z, Shahrooz R, Ahmadi A, Soltanalinejad F (2016). Ethyl Pyruvate Ameliorates The Damage Induced by Cyclophosphamide on Adult Mice Testes. Int J Fertil Steril.

[39] Bradford MM (1976). A rapid and sensitive method for the quantitation of microgram quantities of protein utilizing the principle of protein-dye binding. Anal Biochem.

[40] Shilov IV, Seymour SL, Patel AA, Loboda A, Tang WH, Keating SP, Hunter CL, Nuwaysir LM, Schaeffer DA (2007). The Paragon Algorithm, a next generation search engine that uses sequence temperature values and feature probabilities to identify peptides from tandem mass spectra. Mol Cell Proteomics.

[41] Cai H, Ren Y, Li XX, Yang JL, Zhang CP, Chen M, Fan CH, Hu XQ, Hu ZY, Gao F, Liu YX (2011). Scrotal heat stress causes a transient alteration in tight junctions and induction of TGF-beta expression. Int J Androl.

