# Supplementary material for: Isobaric tags for relative and absolute quantification-based proteomic analysis of testis biopsies in rhesus monkeys treated with transient scrotal hyperthermia
Source: Oncotarget. 2017 Sep 8;8(49):85909–25. doi: 10.18632/oncotarget.20719 (PMC5689656; doi:10.18632/oncotarget.20719)
Supplement: Supplementary file 1 [file oncotarget-08-85909-s001.pdf]

Isobaric tags for relative and absolute quantification-based proteomic analysis of testis biopsies in rhesus monkeys treated with transient scrotal hyperthermia

SUPPLEMENTARY MATERIALS

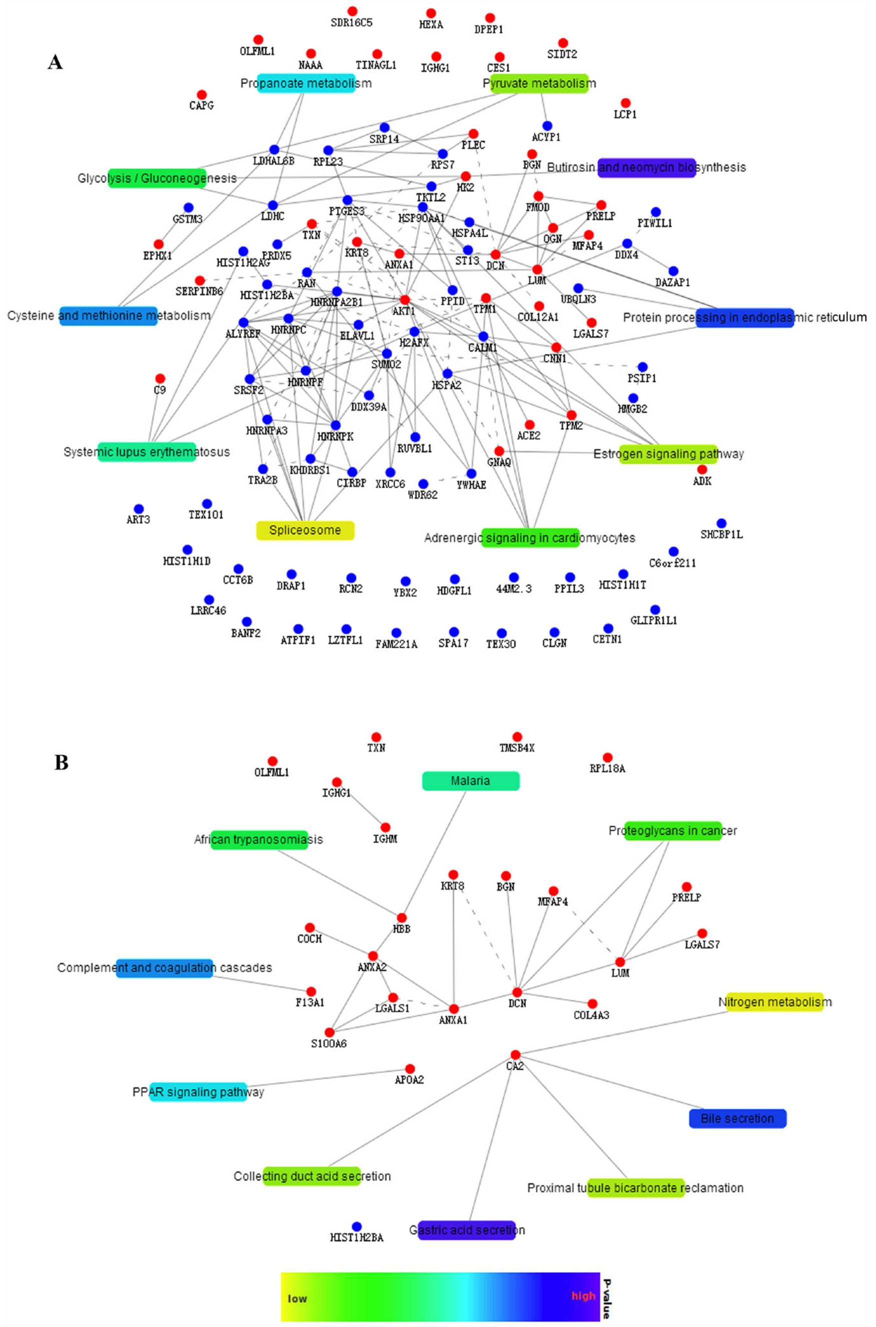

Supplementary Figure 1: PPI network combined with KEGG pathway for differentially expressed proteins identified at day 8 vs day 0 **(A)**, and day 60 and day 0 **(B)**, respectively. Protein interaction analysis was performed against the STRING database. The blue node represents protein that has a negative fold change, the red node represents a positive fold change, and the rectangular node represents a KEGG pathway. The legend shown under the network gives the *p* values of the enriched KEGG pathways. Gradient color is set from yellow to blue (yellow for smaller *p* values, blue for larger *p* values).
